# Reliability and validity estimation of Urdu version of Children Emotion Management Scales (CEMS) in Pakistan

**DOI:** 10.3389/fpsyt.2023.1214708

**Published:** 2024-03-28

**Authors:** Khawer Bilal Baig, Haleema Sadia, Umara Rauf, Qasir Abbas, Zoobia Ramzan, Urooj Tabassum, Sumayah Aljhani

**Affiliations:** ^1^Department of Professional Psychology, Bahria University Lahore Campus, Lahore, Pakistan; ^2^Department of Applied Psychology, Government College University Faisalabad, Faisalabad, Pakistan; ^3^Department of Psychology, Government College Women University Sialkot, Sialkot, Pakistan; ^4^Department of Psychiatry, Dow International Medical College, Dow University of Health Sciences, Karachi, Pakistan; ^5^Department of Psychiatry, College of Medicine, Qassim University, Buraydah, Saudi Arabia

**Keywords:** emotion management, cross-language validation, reliability, exploratory factor analysis, confirmatory factor analysis, validity

## Abstract

**Introduction:**

The present study aimed to translate and validate Children's Emotion Management Scales into Urdu, the national language of Pakistan.

**Method:**

The current study comprised three different phases, i.e., phase I: Cross-language validation over a sample of (*N* = 169) school children, estimated at a 1-week interval.

**Results:**

The results indicate a significant correlation (*r* = 0.846–0.891) at *p* < 0.01. In phase II, the internal consistency reliability (*r* = 0.808–0.904) and split-half reliability (*r* = 0.737–0.898) of the scale were assessed (*N* = 683) at *p* < 0.01. Furthermore, significant results for test-retest reliability analysis (*N* = 168) were obtained (*r* = 0.736–0.917 at *p* < 0.01), following the confirmatory factor analysis (CFA) (*N* = 1,083). Exploratory factor analysis (EFA) was conducted on the same sample chosen for CFA. EFA resulted in the retention of original inhibition (INH), dysregulated expression (DYS), and emotional coping (EMO) factors. CFA findings suggest a good model fit. In phase III, convergent validity and divergent validity were checked (*N* = 385, 255, and 213). Convergent validity of INH and DYS subscales and divergent validity of EMO subscales were established, with SBI (*r* = 0.217–0.609; 0.210–0.445; −0.026 to −0.553), SHS (*r* = 0.417–0.441; 0.480–0.546; −0.338 to −0.582), and suppression subscale of ERQ (*r* = 0.430–0.480; 0.468–0.522; −0.245 to −0.369) at *p <* 0.01. For divergent validity of INH and DYS subscales and convergent validity of EMO subscales, their scores were correlated with the SPS (*r* = −0.204 to −0.350; −0.318 to −0.459; 0.191–0.531), RSE Scale (*r* = −0.226 to −0.351; −0.279 to −0.352; 0.255–0.507), DTS (−0.290 to −0.617; −0.369 to −0.456; 0.246–0.680), and reappraisal subscale of ERQ (*r* = −0.456 to −0.541; −0.329 to −0.544; 0.446–0.601) at *p* < 0.01.

**Discussion:**

It is concluded that the scale is reliable and valid with sound psychometric properties.

## Introduction

Globally, emotional problems are very common in students similar to other mental health issues (1). In Pakistan, behavioral problems are prevalent at an alarming level of 15.9%, with a ratio of 22.5% accounting for emotional problems (2). These problems affect various aspects of life, leading to everyday distress and even severe mental health issues (3). Progressive countries have better management strategies for these problems, but in a country like ours, there is still a long way to go (4). A fairly recent study (5) revealed alarming numbers about the prevalence of depression in the Pakistani population. In their study, Khan et al. (6) significantly report the impact of anger in developing suicidal ideation and depressive symptoms. Sadness and low mood are major diagnostic criteria for depressive disorders (7). Young and Dietrich (8) reported that worry, daily life stress, and rumination accounted for 35% of the variance in anxiety scores and 58% of the variance in depressive symptoms. A meta-analytic review connects unhealthy emotion regulation as a risk factor for various psychopathologies, such as depression, anxiety disorders, eating disorders, and substance use disorders (9).

Emotional regulation is defined as the “extrinsic and intrinsic processes responsible for monitoring, evaluating and modifying emotional reactions, especially their intensive and temporal features, to accomplish one's goals” (10). Emotion regulation is also referred to as a “heterogeneous set of processes” employed to regulate emotions (11). The role of cognitive reappraisal as antecedent-based and expressive suppression as a response or expression-based strategy for emotion management is well defined in the literature (12). Recent studies emphasize the role of healthy relationships including a person's social and intimate relationships, such as parent–child, spousal, and peer relations, as determinants in the adaptive regulation of emotions (13). Literature also strongly advocates the role of child maltreatment as the basis of faulty emotional regulation (14). Many studies (15, 16) have highlighted the importance of emotion regulation, especially with respect to child and adolescent populations. The three main emotions that are usually studied with respect to emotion regulation are anger, worry, and sadness.

Sadness and anger are categorized under seven universal emotions (17). Anger is actively responding to or opposing some event that you do not approve of Kazdin (18). Karnaze and Levine (19) define *Sadness* as an emotion generated when the status or approachability of a predetermined target varies and goal achievement becomes less plausible. While faced with an enemy or life-threatening situation, one can feel anger in response to the distress of this event, or when s/he faces a failure in achieving a target that s/he has predetermined (20). Worry, the third key emotion in the current study, is described as a combination of internal mental images and ideas, subjective understanding, and expressed behavior or overt expression (21). The subscales of Children's Emotion Management Scales (CAMS, CWMS, and CSMS) further consist of items divided into three main categories: inhibition scale items (INH), dysregulated expression scale items (DYS), and emotional coping scale items (EMO). In Joormann and Gotlib (22), inhibition is referred to as a strategy that allows people to stop processing a certain emotion and divert their attention to other aspects of the event that is causing any particular emotion. Emotional dysregulation refers to the “inability to flexibly respond to and manage emotion” (23). Emotional coping can belong to both healthy and adaptive emotional regulation strategies. Coping is “any conscious or non-conscious adjustment or adaptation that decreases tension and anxiety in a stressful experience or situation” (7).

In this study, the role of parenting style, academic burnout, shyness, distress tolerance, and self-esteem have been studied in different capacities as correlates of the main variable to establish its psychometric properties in detail. Burnout in the workplace is described as “a syndrome of emotional exhaustion, cynicism or depersonalization, and reduced professional efficacy” (24). Academic burnout is burnout in the school environment. Walburg (25), in her detailed literature review about burnout in adolescents related to academic activities, amply highlighted the co-occurrence of adolescent psychopathology and academic burnout (26, 27). Shyness is a trait characterized by heightened fear and wariness in new and unknown social situations and self-consciousness and embarrassment in situations of perceived social criticism (28). Shyness is significantly linked with the development of socially anxious behavior in children (29).

It is mandatory to mention the role of parent psychopathology as a determinant factor in a child's mental health. Kerns et al. (30) reported serial mediation, linking maternal anxiety to ineffective emotion regulation during child distress, faulty accommodation of child emotion regulation, and increased anxiety levels in children. Healthy parental care and home environment determine various future aspects of a child's growth, including physical, social, and psychological domains of life (31). A recent study (14) proposed the predictive relationship between parental treatment and emotion regulation tendencies and, ultimately, child psychopathology. Literature suggests that a good sense of self is vital in determining adolescents' healthy emotion management behavior. A recent study (32) proposed the mediating role of self-esteem in the intensity of anxiety and distress traits and emotion regulation ability.

Assessment of emotion regulation in children is an underexplored area of research in Pakistan. Research regarding child emotion, which is mostly concerned with management on behalf of parents or immediate caregivers (33) and children, mainly remains excluded from this process. Another important hindrance in exploring child emotion regulation is the non-availability of appropriate tools in the native language, i.e., Urdu (34). Using scales available in a foreign language is a major cultural barrier to the appropriate assessment and valid findings of the construct being studied (35). The present study aimed to translate and validate Children's Emotion Management Scales. The major goal in translating a scale of a foreign language into a native language is to achieve equivalency. The new scale must have equivalency in four manners (36), namely, linguistic, paradigmatic, stylistic, and textual equivalence. The Children's Emotion Management Scales (CEMS) have been translated into different languages already. Ha and Jue (37) have developed a Korean version of the anger and sadness scales of CEMS. Ogbaselase (38) established the psychometric properties of CEMS on the psychiatric sample.

## Methods

This research study was completed in three different phases following the standard translation–validation process. All phases adhered to the transparency and openness protocols stated below.

### Transparency and openness

We followed the Journal of Applied Psychology methodological checklist to describe our sampling plan, all data exclusions (if any), all manipulations, and all measures in the study. All data and other related research materials are available at (Link will be provided later). Data were analyzed using SPSS version 26 and AMOS version 23. The study design and analysis were not preregistered.

### Phase I: cross-language validation

#### Brief description of scale

The Children's Emotion Management Scales (CEMS) have been translated into Urdu language (15, 16). The CEMS comprises 33 items that assess the regulation of emotions in three major domains, namely, anger (11 items), worry (10 items), and sadness (12 items). Each domain is subdivided into three categories, i.e. (i) inhibition, (ii) dysregulated expression, and (iii) emotional coping. The CEMS has a 3-point Likert-type response scale. Rating values of Cronbach's alpha for the three major domains are between 0.62 and 0.77, and test–retest reliability ranges from 0.61 to 0.30, respectively. Initial norms of the scales were developed in children from 9 to 12 years of age.

#### Expert panel

Following the standard procedure of translation–validation (39), a panel of four experts was formed. These experts thoroughly investigated the forward and backward translation versions of the scale; after which, the final drafting was carried out for linguistic validation.

#### Selection of expert translators

It is very important for the authenticity of the translation procedure that the translators are expert and skilled with complete knowledge of test translation (40). Translators appointed for forward and backward translations were experts in both the source and target language, which in the present case were English and Urdu, respectively.

#### Forward translation

The first part of the standard translation procedure is to do a forward translation of the original scale in the language of choice, which in the present case is Urdu. A group of four bilingual experts were asked to translate the scales into the Urdu language. Forward translations of scales were obtained individually. Later, the first draft for backward translation was finalized from these translations, applying a reconciliation mechanism, whereby different individual translations are observed and merged into a single draft (41).

#### Backward translation

Backward translation is a necessary step in scale translation; without backward translation, contextual and semantic equivalence cannot be achieved (42). The final draft of the forward translation was presented before a panel of bilingual experts. Similar to forward translations, backward translations were obtained individually. The panel of experts thoroughly compared these translations with the original scales and looked for contextual, semantic, and overall similarities, following which they showed unanimous confidence in the obtained Urdu version of the scales. Then, this final draft was selected for cross-language validation of scales.

#### Cross-language validation

In this phase, the cross-language validation of the scale proceeded.

**Sample:** A total of 169 participants took part in this phase. This total sample was further divided into three samples; these subsamples were used for cross-language validation of each of the subscales of the Children's Emotion Management Scales, namely, sample A, sample B, and sample C for Children's Anger Management Scales (CAMS), Children's Worry Management Scale (CWMS), and Children's Sadness Management Scales (CSMS), respectively. The age range of the samples was 10 to 18 years, with the mean age for CAMS = 14.43, CWMS = 13.53, and CSMS = 13.68 years.

Sample A consisted of 81 participants, with 54 (66.7%) boys and 27 (33.3%) girls. Sample B consisted of 38 participants, with 23 (60.5%) boys and 15 (39.5%) girls, and sample C consisted of 50 girls. In sample A, 30 children were enrolled from 6th grade, 8 from 7th grade, 15 from 8th grade, 4 from 9th grade, and 24 from 10th grade. In sample B, 13 children were enrolled from 7th grade and 25 from 8th grade. In sample C, all children were students of 8th grade. For samples A, B, and C, the average class attendance was 85.38%, 86.73%, and 91.20% and the average daily study hours were 4.4, 3.74, and 5.20, respectively. Overall, the academic grades ranged from A+ to D; A+ (*N* = 20, 8, 0), A (*N* = 24, 2, 21), B (*N* = 19, 19, 24), C (*N* = 16, 8, 4), and D (*N* = 2, 1, 1).

**Procedure:** Both versions of the scales, i.e., the original CEMS and the newly translated Urdu version of CEMS, were administered to these samples, and the scores were correlated to determine the similarity between the original and adapted versions.

Findings show (Table 1) significant linguistics equivalence for CEMS. Correlation coefficient statistics values for CAMS, CWMS, and CSMS are 0.846, 0.851, and 0.911, which indicate significant correlation. For individual items, these values ranged from 0.714 to 0.936 for CAMS, from 0.768 to 0.938 for CWMS, and 0.720 to 0.948 for CSMS. Moreover, for test–retest reliability analysis, Pearson's correlation values *(r)* for CAMS, CWMS, and CSMS are 0.869, 0.881, and 0.917, respectively. For individual items, these values ranged from 0.639 to 0.986 for CAMS, from 0.750 to 0.930 for CWMS, and 0.714 to 0.888 for CSMS. This indicates that all three domains yield excellent cross-language equivalence.

**Table 1:** Linguistic equivalence (LE) and test–retest reliability (TRT) values of children's emotion management scales (CEMS).

	**CAMS**		**CWMS**		**CSMS**	
**S. no**.	**LE (*N =* 81)**	**TRT (*N =* 52)**	**LE (*N =* 38)**	**TRT (*N =* 57)**	**LE (*N =* 50)**	**TRT (*N =* 59)**
1	0.770^**^	0.639^**^	0.938^**^	0.815^**^	0.948^**^	0.888^**^
2	0.904^**^	0.877^**^	0.836^**^	0.843^**^	0.814^**^	0.751^**^
3	0.883^**^	0.921^**^	0.912^**^	0.788^**^	0.781^**^	0.758^**^
4	0.936^**^	0.947^**^	0.904^**^	0.811^**^	0.880^**^	0.844^**^
5	0.836^**^	0.986^**^	0.884^**^	0.784^**^	0.795^**^	0.748^**^
6	0.796^**^	0.937^**^	0.768^**^	0.750^**^	0.870^**^	0.858^**^
7	0.866^**^	0.929^**^	0.901^**^	0.867^**^	0.755^**^	0.714^**^
8	0.917^**^	0.986^**^	0.872^**^	0.794^**^	0.720^**^	0.764^**^
9	0.856^**^	0.898^**^	0.868^**^	0.930^**^	0.844^**^	0.811^**^
10	0.714^**^	0.935^**^	0.816^**^	0.779^**^	0.809^**^	0.798^**^
11	0.912^**^	0.983^**^	-	-	0.769^**^	0.733^**^
12	-	-	-	-	0.839^**^	0.808^**^
Total	0.846^**^	0.869^**^	0.851^**^	0.881^**^	0.911^**^	0.917^**^
INH	0.891^**^	0.939^**^	0.967^**^	0.807^**^	0.904^**^	0.861^**^
DYS	0.912^**^	0.943^**^	0.927^**^	0.917^**^	0.761^**^	0.781^**^
EMO	0.885^**^	0.907^**^	0.907^**^	0.736^**^	0.897^**^	0.877^**^

^**^p > 0.01; 1 week interval CAMS, Children's Anger Management Scale; CWMS, Children's Worry Management Scale; CSMS, Children's Sadness Management Scale.

### Phase II – psychometric evaluation of Urdu children emotion management scales

#### Internal consistency reliability

In this phase, first of all, the internal consistency of the scale was checked to ensure whether the adapted scale could proceed to further stages or not. This internal consistency reliability indicates scale yield outstanding reliability estimation.

For this purpose, a sample of *N* = 683 children was selected, including 347 boys and 336 girls. In the sample, 98 children were enrolled in 6th grade, 115 in 7th grade, 248 in 8th grade, 149 in 9th grade, and 73 in 10th grade. The mean age for this sample was 13.74 years. Further demographic information on this sample is provided in **Table 7**. The sample was selected from different schools in Lahore and Faisalabad using a convenient sampling technique.

**Procedure:** Cronbach's alpha is a significant measure of establishing the internal consistency reliability of a scale (43). The value of Cronbach's alpha was determined for the translated scales to estimate internal consistency. A higher value of Cronbach's α closer to 1 indicates stronger internal consistency (44).

Furthermore, the reliability estimation and split-half reliability were cross-checked which indicates that the scale has sound reliabilities (Table 2). For example, the reliability estimation of INH of three scales was estimated at 0.867, 0.871, and 0.891 with SHR estimation of 0.864, 0.857, and 0.898. Similarly, the DYS subscale of anger, worry, and sadness reliability estimation was 0.904, 0.808, and 0.889 with SHR estimated at 0.766, 0.737, and 0.781 respectively. The reliability estimation of the EMO subscale of anger, worry, and sadness reliability was estimated at 0.895, 0.822, and 0.836 with SHR estimation of 0.888, 0.745, and 0.783, respectively.

**Table 2:** Cronbach's alpha (α) and split half reliability (SHR) values for translated children's emotion management scales (*N* = 683).

	**CAMS**		**CWMS**		**CSMS**	
**Subscales**	**α**	**SHR**	**α**	**SHR**	**α**	**SHR**
INH	0.867^**^	0.864^**^	0.871^**^	0.857^**^	0.891^**^	0.898^**^
DYS	0.904^**^	0.766^**^	0.808^**^	0.737^**^	0.889^**^	0.781^**^
EMO	0.895^**^	0.888^**^	0.822^**^	0.745^**^	0.836^**^	0.783^**^

^**^p > 0.01; CAMS, Children's Anger Management Scale; CWMS, Children's Worry Management Scale; CSMS, Children's Sadness Management Scale; INH, Inhibition Subscale; DYS, Dysregulated Expression Subscale; EMO, Emotional Coping Subscale.

#### Exploratory factor analysis

**Sample:** A collective sample of *N* = 1,083 adolescents, including both boys and girls, who were enrolled in grades 6-10 (at the time of initiation of this study), was selected from different schools of Faisalabad city, using a convenient sampling technique for confirmatory factor analysis (sample details discussed under Confirmatory Factor Analysis section). Exploratory factor analysis was conducted on the sample selected for CFA.

**Procedure:** EFA was conducted using the principal component extraction method. Kaiser–Meyer–Olkin (KMO) measure of sampling adequacy and Bartlett's test of sphericity were used to check the practical suitability of the sample. KMO value >0.70 and selecting factor Eigen value >1 indicate factor significance and test adequacy (45). After establishing sampling adequacy, the factor exploration was completed using the Varimax rotation method.

Before the EFA, Kaiser–Meyer–Olkin (KMO) measure of sampling adequacy and chi-square (χ ^2^) values were obtained. Significant KMO values ranging from 0.63 to 0.80 were obtained for CAMS, from 0.67 to 0.82 for CWMS, and 0.68 to 0.82 for CSMS, respectively. The χ ^2^ values from Bartlett's test performed were also significant, thus ensuring sampling adequacy. The EFA was performed using varimax rotation, resulting in the formation of rotated component matrices for the three subscales (Table 3).

**Table 3:** Kaiser–Meyer–Olkin (KMO) measure of sampling adequacy and chi-square (χ^2^) values for Urdu inhibition, dysregulated expression, and emotional coping subscales and their totals, for subscales and total of children's emotion management scales.

**Subscales**	**CAMS**		**CWMS**		**CSMS**		**CEMS**	
	**KMO**	***χ^2^***	**KMO**	***χ^2^***	**KMO**	***χ^2^***	**KMO**	***χ^2^***
INH	0.78	1586.71	0.72	1722.09	0.80	1744.58	0.77	5213.34
DYS	0.63	380.03	0.68	785.43	0.68	803.70	0.67	2016.13
EMO	0.73	613.85	0.67	784.79	0.82	1228.64	0.76	2763.13
Total	0.80	2971.96	0.82	4023.15	0.80	2971.96	0.84	12269.46

^**^p > 0.01; χ^**2**^ = Chi-Square; INH, Inhibition Subscale; EMO, Emotional Coping Subscale; DYS, Dysregulated Emotion Expression Subscale.

Exploratory factor analysis retained the original factors of all the subscales of CEMS (CAMS, CWMS, and CSMS), as displayed in **Table 7**. For CAMS, Factor 1 to load, comprised all the items of the INH subscale, with the loading sequence AI-5, AI-2, AI-7, and AI-11 and loading values ranging from 0.751 to 0.835. Factor 2 to load, comprised all the items of the EMO subscale, with the loading sequence AE-8, AE-1, AE-10, and AE-3, and loading values ranging from 0.666 to 0.754. Factor 3 to the load consisted of all the items of the DYS subscale, with loading sequence AD-6, AD-9, and AD-4, and loading values ranging from 0.650 to 0.779.

For CWMS, Factor 1 to load comprised all the items of the INH subscale, with the loading sequence WI-6, WI-2, WI-8, and WI-3, and loading values ranging from 0.707 to 0.847. Factor 2 to load consisted of all the items of the DYS subscale, with the loading sequence WD-7, WD-9, and WD-5, and loading values ranging from 0.697 to 0.828. Factor 3 to load comprised all the items of the EMO subscale, with the loading sequence WE-10, WE-1, and WE-4, and loading values ranging from 0.792 to 0.810.

For CSMS, Factor 1 to load comprised all the items of the INH subscale, with loading sequence SI-2, SI-5, SI-7, and SI-12, and loading values ranging from 0.591 to 0.861. Factor 2 to load comprised all items of the EMO subscale, with loading sequence SE-8, SE-1, SE-6, SE-10, and SE-3, and loading values ranging from 0.669 to 0.738. Factor 3 to load consisted of all items of DYS, with loading sequence SD-4, SD-11, and SD-9, and loading values ranging from 0.759 to 0.769. Values of the rotated component matrix show the strength of correlation and consistency between the scale items and subscales (Table 4).

**Table 4:** Rotated component matrix for overall items of Urdu children emotion management scales using Varimax rotation method (*N* = 1,083).

		**CAMS**			**CWMS**			**CSMS**				
		**Factors**			**Factors**			**Factors**				
	**Items**	**1**	**2**	**3**	**Items**	**1**	**2**	**3**	**Items**	**1**	**2**	**3**
	AI-5	0.835			WI-6	0.847			SI-2	0.861		
	AI-2	0.778			WI-2	0.719			SI-5	0.829		
	AI-7	0.778			WI-8	0.710	0.302		SI-7	0.805		
	AI-11	0.751			WI-3	0.707			SI-12	0.591		0.359
	AE-8		0.754		WD-7		0.828		SE-8		0.738	
	AE-1		0.709		WD-9		0.783		SE-1		0.730	
	AE-10		0.675		WD-5	0.324	0.697		SE-6		0.679	
	AE-3		0.666		WE-10			0.810	SE-10		0.673	
	AD-6			0.779	WE-1			0.805	SE-3		0.669	
	AD-9			0.768	WE-4			0.792	SD-4			0.769
	AD-4			0.650	-	-	-	-	SD-11			0.760
	-	-	-	-	-	-	-	-	SD-9			0.759
Eigen values		3.480	1.629	1.298		4.109	1.657	0.895		4.660	1.682	1.085
% of variance		31.635	14.805	11.799		41.094	16.567	8.953		38.837	14.015	9.038
Accumulative %		31.635	46.440	58.239		41.094	57.661	66.615		38.837	52.852	61.890

^**^p > 0.01; Values < 0.30 are suppressed; CAMS, Children's Anger Management Scale; CWMS, Children's Worry Management Scale; CSMS, Children's Sadness Management Scale; AI, Anger Inhibition Subscale; AE, Anger Emotional Coping Subscale; AD, Anger Dysregulated Emotion Expression Subscale; WI, Worry Inhibition Subscale; WD, Worry Dysregulated Emotion Expression Subscale; WE, Worry Emotional Coping Subscale; SI, Sadness Inhibition Subscale; SE, Sadness Emotional Coping Subscale; SD, Sadness Dysregulated Emotion Expression Subscale.

#### Test–retest reliability

**Sample:** A total of 168 girls were included in the sample to check the linguistic equivalence of translated versions of Children's Emotion Management Scales (CEMS) from different schools in Lahore and Faisalabad. The sample was further into three subsamples, namely, sample A (*N* = 57), B (*N* = 52), and C (*N* = 59) for the Children's Anger Management Scale, the Children's Worry Management Scale, and the Children's Sadness Management Scale, respectively. The age range of the sample was 12 to 16 years, with the mean age for CAMS = 13.67, CWMS = 13.71, and CSMS = 13.64 years. All the children were enrolled in 8th grade. The children in samples A, B, and C had an average class attendance of 91.81, 91.63, and 91.61%, an average of 4.68, 4.83, and 4.76 daily study hours, respectively, with Academic Grades from A to C; A (*N* = 21, 20, 21), B (*N* = 31, 28, 33), and C (*N* = 5, 4, 5).

**Procedure:** Test–retest reliability refers to the consistency in test scores when administered to the same individual in two different instances. Test–retest reliability was determined to ensure the reliability of scales further. A strong correlation between the scores of the two administrations will reveal a strong test–retest reliability (46).

**Results:** Table 5 shows that Cronbach's alpha values for test–retest reliability of 0.939^**^ for the CAMS Inhibition Subscale, 0.943^**^ for the CAMS Dysregulated Expression Subscale, 0.907^**^ for the CAMS Emotional Coping Subscale, 0.807^**^ for the CWMS Inhibition Subscale, 0.917^**^ for the CWMS Dysregulated Expression Subscale, 0.736^**^ for the CWMS Emotional Coping Subscale, 0.861^**^ for the CSMS Inhibition Subscale, 0.781^**^ for the CSMS Dysregulated Expression Subscale, and 0.877^**^ for CSMS Emotional Coping Subscale. Test–retest reliability estimation of all subscales indicates scale has sound test–retest reliability, which is suitable for further use.

**Table 5:** Test retest reliability of inhibition, dysregulated expression and emotional coping subscales for three main subscales of children emotion management scales.

**Subscales**	***Mean***	***SD***	**α**	***N***
CAMS – INH-U	7.912	1.805	0.939^**^	52
CAMS – INH-E	7.912	1.795		
CAMS – DEX-U	4.245	1.073	0.943^**^	
CAMS – DEX-E	4.280	1.130		
CAMS – EMC-U	8.456	1.964	0.907^**^	
CAMS – EMC-E	8.596	1.869		
CWMS – INH-U	8.923	1.544	0.807^**^	57
CWMS – INH-E	8.923	1.724		
CWMS – DEX-U	6.192	1.657	0.917^**^	
CWMS – DEX-E	6.288	1.871		
CWMS – EMC-U	6.711	1.257	0.736^**^	
CWMS – EMC-E	6.961	1.533		
CSMS – INH-U	9.000	1.884	0.861^**^	59
CSMS – INH-E	9.118	2.060		
CSMS – DEX-U	5.796	1.483	0.781^**^	
CSMS – DEX-E	5.694	1.567		
CSMS – EMC-U	11.186	2.330	0.877^**^	
CSMS – EMC-E	10.559	2.276		

1 week interval for test-retest reliability analysis; CAMS, Children's Anger Management Scale; CWMS, Children's Worry Management Scale; CSMS, Children's Sadness Management Scale; INH, Inhibition Subscale Items; DEX, Dysregulated Expression Subscale Item; EMC, Emotional Coping Subscale Items; U, Urdu version; E, English version. ^*^p < 0.01, ^**^p < 0.001, ^***^p < 0.0001.

#### Confirmatory factor analysis

**Sample:** The sample for CFA consisted of *N* = 1, 083 children including 547 boys and 536 girls. In the sample, 140 children were enrolled in 6th grade, 212 in 7th grade, 327 in 8th grade, 331 in 9th grade, and 73 in 10th grade. The mean age for this sample was 13.90 years. Further demographic information on this sample is provided in **Table 7**. The sample was selected from different schools in Lahore and Faisalabad using a convenient sampling technique.

**Figure 1:**
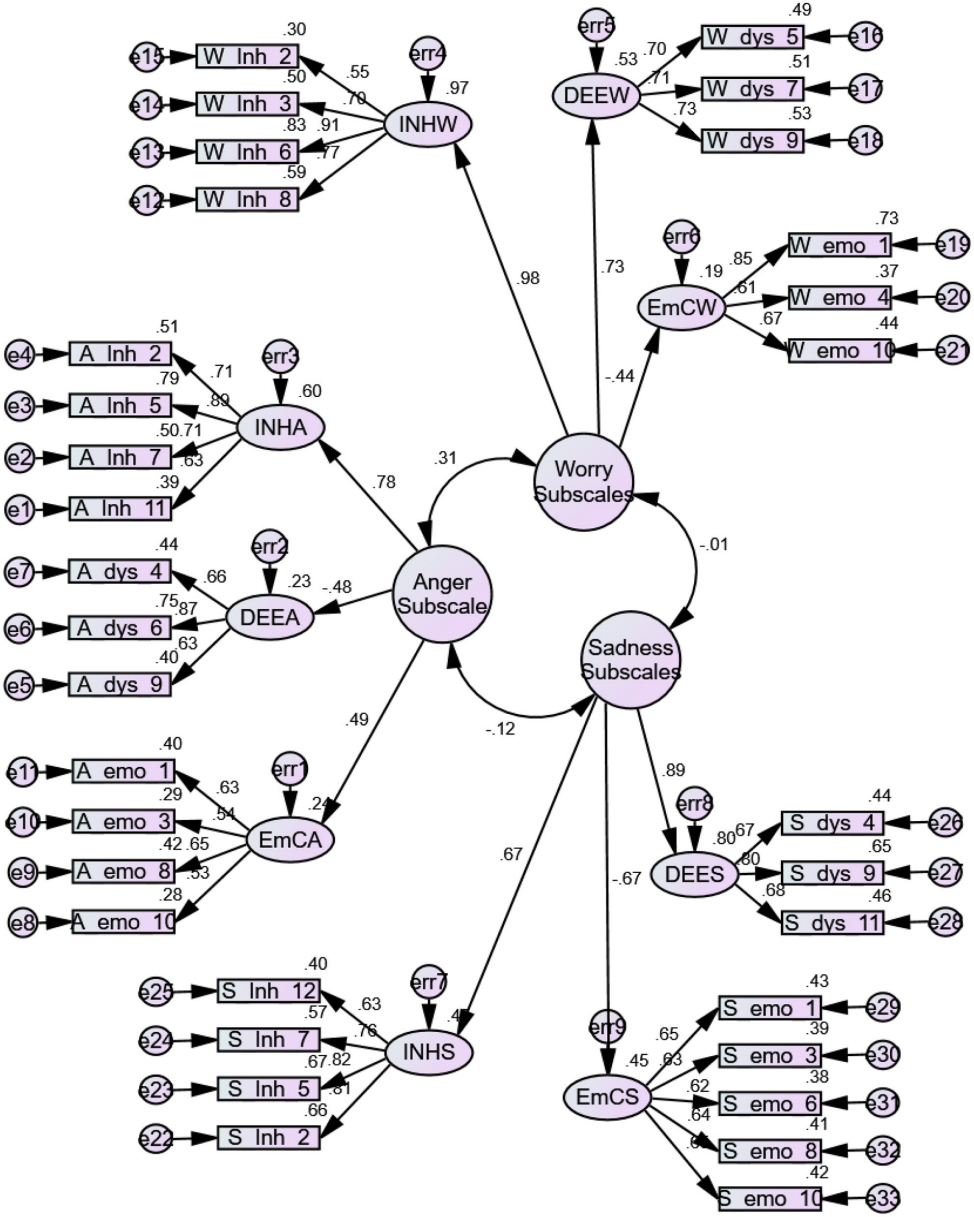
Measurement model of children emotion management scale (children).

**Procedure:** Confirmatory factor analysis (CFA) was conducted to estimate the goodness of fit for the translated scales. CFA was performed using IBM SPSS Amos 21 software. Different model fit indices were estimated. Significant root mean square error of approximation (RMSEA) values indicate good model fit (Figure 1).

## Results

Confirmatory factor analysis (Table 6) is a reliable measure for establishing the reliability and consistency of a standardized psychological measure (47). The data obtained from the confirmatory factor analysis for the present study revealed significant results required for a good model fit. A root mean square error of approximation (RMSEA) of 0.01, 0.05, and 0.08–1 indicates excellent, good, and mediocre model fitness (48). Table 6 shows that RMSEA values for three subscales, CAMS, CWMS, and CSMS, were 0.046, 0.065, and 0.060, respectively, indicating a good model fit. Other model fit indices like goodness-of-fit Index (GFI) and adjusted GFI also had significant values for the three subscales; a value closer to 1 indicates a good model fit (49). Normative Fit Index, Relative Fit Index, Tucker Lewis Index, and Comparative Fit Index also support the consistency and model fitness of Children's Emotion Management Scales (CEMS).

**Table 6:** Values for different indices of model fit in confirmatory factor analysis.

	**χ^2^**	**df**	**GFI**	**AGFI**	**NFI**	**RFI**	**TLI**	**RMSEA**	**CFI**
CAMS	136.003	41	0.978	0.964	0.954	0.939	0.956	0.046	0.968
CWMS	180.255	32	0.968	0.945	0.955	0.937	0.948	0.065	0.963
CSMS	249.628	51	0.963	0.943	0.946	0.930	0.943	0.060	0.956
CEMS	1310.28	483	0.931	0.920	0.898	0.888	0.927	0.040	0.933

### Phase III – validity estimates of Urdu CEMS

#### Brief descriptions of the scales

For Validation estimates of the translated scales, the following measures were used.

**School burnout inventory (SBI)** (50)**:** A translated version of the original School Burnout Inventory was developed for use in the study. SBI is a 10-item scale, with a Likert-type response scale ranging from 1 to 6, with 1 being “Completely Agree” and 6 being “Completely Disagree.” It is divided into three subscales, namely, Exhaustion (four items), Inadequacy (three items), and Cynicism (three items). Cronbach's alpha of SBI is 0.88.

**Shyness scale (SS)** (51)**:** A translated version of the original Shyness Scale was developed for use in the study. SS is a 14-item questionnaire, with a Likert-type response scale ranging from 1 to 5, with 1 being “Strongly Disagree” and 5 being “Strongly Agree.” Cronbach's alpha value of the Shyness Scale is 0.94.

**Emotion regulation questionnaire** (52)**:** A translated version of the original scale of the Emotion Regulation Questionnaire (ERQ) was developed for use in the study. It is a 10-item questionnaire, with a Likert-type response scale ranging from 1 “Strongly Disagree” to 7 “Strongly Agree.” Cronbach's alpha estimate of the Emotion Regulation Questionnaire gives a value between 0.76 and 0.90.

**Scale of parenting style** (53)**:** A translated version of the original Scale of Parenting Style was developed for use in the study. The scale of Parenting Style is a 38-item questionnaire, with a Likert-type response scale ranging from 1 to 5, with 1 being “Very Wrong” and 5 being “Very Right.” The test–retest reliability of the Scale of Parenting Style after 1 week ranged between 0.81 and 0.83.

**Rosenberg self-esteem scale** (54)**:** The translated version of the Rosenberg Self-Esteem Scale was used in this study. RSE is a 10-item self-report questionnaire, with a Likert-type response scale, ranging from 1 to 4, with 1 being “Strongly Disagree” to 4 being “Strongly Agree.” Items 2, 5, 6, 8, and 9 are reverse coded. The aggregate of total items shows the level of self-esteem; the higher the score the greater the level of self-esteem. Cronbach's alphas for RSE are in the range of 0.77 to 0.88.

**Distress tolerance scale:** (55)**:** A translated version of the Distress Tolerance Scale (DTS) was used in the study. It is a 16-item self-report measure with a 5-point Likert-type rating scale, with 1 being “Strongly agree,” 2 being “Mildly agree,” 3 being “Agree and disagree equally,” 4 being “Mildly disagree,” and 5 being “Strongly disagree.” Cronbach's alpha for DTS is 0.89.

#### Establishing convergent and divergent validity

**Sample: Three** different samples were selected from different schools in Lahore and Faisalabad. The age range of the sample was 10 to 18 years, with the mean ages for the three samples as 14.17, 13.85, and 13.19 years, respectively. Sample 1 consisted of 385 children with 193 (50.13%) boys and 192 (49.87%) girls, respectively; sample 2 consisted of 255 children with 119 (46.67%) boys and 136 (53.3%) girls; and sample 3 consisted of 213 children with 89 (41.78%) boys and 124 (58.22%) girls. In sample 1, 42 children were enrolled in 6th grade, 96 in 7th grade, 76 in 8th grade, and 171 in 9th grade. In sample 2, 67 children were enrolled in 6th grade, 33 in 7th grade, 10 in 8th grade, 105 in 9th grade, and 40 in 10th grade. In sample C, 53 children were enrolled in 6th grade, 57 in 7th grade, 55 in 8th grade, and 48 in 9th grade.

**Procedure:** To establish the convergent and divergent validities of the translated version of Children's Emotion Management Scales, multiple correlational analyses were performed.

**Results:** Multiple correlational analyses were conducted to establish convergent validity of inhibition subscales of CAMS, CWMS, and CSMS. Results of correlational analysis with shyness were 0.424, 0.441, and 0.417, and with suppression subscales of ERQ were 0.440, 0.430, and 0.480 for CAMS, CWMS, and CSMS, respectively. The values of correlation of the inhibition scale with the cynicism subscale were 0.273, 0.231, and 0.300 with the exhaustion subscale were 0.217, 0.388, and 0.249 and with the inadequacy subscale were 0.609, 0.446, and 0.45 for CAMS, CWMS, and CSMS, respectively. Multiple correlational analyses were conducted to establish the convergent validity of dysregulated expression subscales of CAMS, CWMS, and CSMS. Correlational analysis with shyness revealed 0.546, 0.500, and 0.480, and with the suppression subscale of ERQ were 0.522, 0.518, and 0.468 for CAMS, CWMS, and CSMS, respectively. With the cynicism subscale, the values of *r* were 0.370, 0.326, and 0.361, with the exhaustion subscale, were 0.261, 0.210, and 0.332 and with the inadequacy subscale were 0.386, 0.380, and 0.445 for CAMS, CWMS, and CSMS, respectively (Table 7).

**Table 7:** Validity estimates of translated CEMS with different standardized psychological measures.

	**DTS (*N =* 385)**	**PSS (*****N =*** **255)**		**RSE (*N =* 255)**	**ERQ (*****N =*** **179)**		**SS (*N =* 213)**	**BOI (*****N =*** **255)**		
		**SPS-F**	**SPS-M**		**REA**	**SUP**		**INA**	**CYN**	**EXH**
A-INH	−0.527^**^	−0.350^**^	−0.216^**^	−0.351^**^	−0.468^**^	0.440^**^	0.424^**^	0.609^**^	0.273^**^	0.217^**^
W-INH	−0.290^**^	−0.209^**^	−0.204^**^	−0.226^**^	−0.456^**^	0.430^**^	0.441^**^	0.446^**^	0.231^**^	0.388^**^
S-INH	−0.617^**^	−0.253^**^	−0.208^**^	−0.268^**^	−0.541^**^	0.480^**^	0.417^**^	0.450^**^	0.300^**^	0.249^**^
A-DYS	−0.456^**^	−0.305^**^	−0.459^**^	−0.279^**^	−0.544^**^	0.522^**^	0.546^**^	0.386^**^	0.370^**^	0.261^**^
W-DYS	−0.403^**^	−0.346^**^	−0.348^**^	−0.352^**^	−0.329^**^	0.518^**^	0.500^**^	0.380^**^	0.326^**^	0.210^**^
S-DYS	−0.369^**^	−0.318^**^	−0.333^**^	−0.350^**^	−0.487^**^	0.468^**^	0.480^**^	0.445^**^	0.361^**^	0.332^**^
A-EMO	0.246^**^	0.460^**^	0.531^**^	0.507^**^	0.446^**^	−0.245^**^	−0.582^**^	−0.198^**^	−0.553^**^	−0.176^**^
W-EMO	0.680^**^	0.342^**^	0.371^**^	0.339^**^	0.601^**^	−0.349^**^	−0.479^**^	−0.184^**^	−0.315^**^	−0.171^**^
S-EMO	0.354^**^	0.234^**^	0.191^**^	0.255^**^	0.595^**^	−0.369^**^	−0.338^**^	−0.089	−0.222^**^	−0.026

^**^p > 0.01; A-INH, inhibition subscale of Children's Anger Management Scale (CAMS); W-INH, inhibition subscale of Children's Worry Management Scale (CWMS); S-INH, inhibition subscale of Children's Sadness Management Scale (CSMS); A-DYS, dysregulated expression subscale of CAMS; W-DYS, dysregulated expression subscale of CWMS; S-DYS, dysregulated expression subscale of CSMS; A-EMO, emotional coping subscale of CAMS; W-EMO, emotional coping subscale of CWMS; S-EMO, emotional coping subscale of CSMS; DTS, Distress Tolerance Scale; PSS, Scale of Parenting Style (SPS-F, SPS Father and SPS-M, SPS Mother); RSE, Rosenberg Self-Esteem Scale; ERQ, Emotion Regulation Questionnaire (REA, reappraisal subscale and SUP, suppression subscale); SS, Shyness Scale; SBI, School Burnout Inventory (INA, inadequacy subscale, CYN, cynicism subscale and EXH, exhaustion).

Multiple correlational analyses were conducted to establish divergent validity of inhibition subscales of CAMS, CWMS, and CSMS. With reappraisal subscale, the values for *r* were observed as −0.468, −0.456, and −0.541, and with DTS *r* values were −0.527, −0.290, and −0.617 (*p* < 0.01) for CAMS, CWMS, and CSMS, respectively. With PSS, the correlation analysis resulted in r values of −0.350, −0.209, and −0.253 for the SPS father subscale, −0.216, −0.204, and −0.208 for the SPS mother subscale, and with Rosenberg self-esteem generated values of *r* were −0.351, −0.226, and −0.268 for CAMS, CWMS, and CSMS, respectively. Similar correlational analyses were performed to establish divergent validity of dysregulated expression subscales of CAMS, CWMS, and CSMS. With the reappraisal subscale, the values for *r* were −0.544, −0.329, and −0.487, and with DTS, *r* values were −0.456, −0.403, and −0.369 for CAMS, CWMS, and CSMS, respectively. With PSS, the correlation analysis resulted in r values of −0.305, −0.346, and −0.318 for the SPS father subscale and −0.459, −0.348, and −0.333 for the SPS mother subscale (*p* < 0.01) for CAMS, CWMS, and CSMS. Correlation analysis with Rosenberg self-esteem generated values of *r* were −0.279, −0.352, and −0.350 for CAMS, CWMS, and CSMS, respectively (Table 7).

Multiple correlational analyses were conducted to establish the convergent validity of emotional coping subscales of CAMS, CWMS, and CSMS. With the reappraisal subscale of ERQ, the values for *r* were observed as 0.446, 0.601, and 0.595, and with DTS *r* values were 0.246, 0.680, and 0.354 for CAMS, CWMS, and CSMS, respectively. With PSS, the correlation analysis resulted in *r* values 0.460, 0.342, and 0.234 for the SPS father subscale, 0.531, 0.371, and 0.191 for the SPS mother subscale, and with Rosenberg self-esteem generated values of *r* were 0.507, 0.339, and 0.255 for CAMS, CWMS, and CSMS, respectively. Multiple correlational analyses were conducted to establish divergent validity of emotional coping subscales of CAMS, CWMS, and CSMS. The *r* values with the cynicism subscale were −0.553, −0.315, and −0.222, with the exhaustion subscale, were −0.176, −0.171 and with the inadequacy subscale were −0.198, −0.184, and −0.089 for CAMS, CWMS, and CSMS, respectively. With ERQ, the *r* values were −0.245, −0.349, and −0.369 for CAMS, CWMS, and CSMS, respectively. With shyness, *r* values were −0.582, −0.479, and −0.338 for CAMS, CWMS, and CSMS, respectively (Table 7).

Furthermore, different samples were collected at different stages of the study. In each sample, only participants they completed the whole testing procedures and successfully completed all assessment measures. Participants with any history of mental health problems/disability were excluded from the sample. Some participants were excluded due to a variety of reasons, and they are mentioned in Table 8 with the category of discarded forms before the total sample. For example, the discarded forms were as in samples of LE = 9, TR = 5, ICR = 16, CFA = 31, sample 1 = 20, sample 2 = 11, and sample 3=8 forms, respectively (Table 8). Discarded forms were not included in the data analysis.

**Table 8:** Demographic information of the samples used in phase I, II, and III of the study.

**Demo-Graphics**	**Categories**	**Phase I**		**Phase II**		**Phase III**		
		**Sample LE (*N =* 169)**	**Sample TR (*N =* 168)**	**Sample ICR (*N =* 683)**	**Sample CFA (*N =* 1,083)**	**Sample 1 (*N =* 385)**	**Sample 2 (*N =* 255)**	**Sample 3 (*N =* 213)**
Gender	Male	77	-	347	547	193	119	89
	Female	92	168	336	536	192	136	124
Class	6th	30	-	98	140	42	67	53
	7th	21	-	115	212	96	33	57
	8th	90	168	248	327	76	10	55
	9th	4	-	149	331	171	105	48
	10th	24	-	73	73	-	40	-
School	Government	65	168	38	398	146	133	34
Structure	Private	104	-	645	685	239	122	179
Father	Govt. Job	14	84	263	315	335	68	73
Occupation	Private Job	103	78	357	688	50	187	140
	Other	52	13	-	80	-	-	-
Mother	Housewife	150	168	651	1,051	385	236	207
Occupation	Working Woman	19	-	32	32	-	19	6
Family Structure	Joint	88	107	393	552	152	125	94
	Nuclear	81	61	290	531	233	130	119
Age	Mean	14.01	13.67	13.74	13.9	14.17	13.85	13.19
	SD	1.518	0.844	1.587	1.737	1.94	1.231	1.478
Discarded Forms		9	5	16	31	20	11	8
Total sample		178	173	699	1,114	405	266	221

LE, Language Equivalence; TR, Test retest Reliability; ICR, Internal Consistency Reliability; CFA, Confirmatory Factor Analysis.

## Discussion

The present study aimed to translate and validate Children's Emotion Management Scales (CEMS) (15, 16). Phase I of the study was cross-language validation. The panel of bilingual experts was asked to forward and then backward translate the scale. After back-translation, a final version of the scale was prepared for cross-language validation. Cross-language validation was achieved by estimating correlations between the subscales of the Urdu version and the original version of the main scales conducted on three samples (one for each subscale) in two administrations timed at a one-week interval. A higher value of Cronbach's alpha closer to 1.00 indicates stronger reliability. The results reported significant reliability values, thus ensuring linguistic equivalence. The next phase of the study was to perform multiple reliability analyses to ensure the psychometric soundness of the translated version of Children's Emotion Management Scales (CEMS) as per the standard translation validation procedure.

Reliability analyses were performed using IBM SPSS software. Cronbach's alpha value represents the internal soundness and consistency of a psychological measure (56). The same sample, on which the internal consistency reliability was estimated, was used to estimate split-half reliabilities. The Cronbach's alpha values for the Korean version were 0.75 (inhibition) and 0.59 (dysregulated expression) for CSMS and 0.77 and 0.64 for CAMS (37). The present analysis generated significantly better values than the translated Korean version. Before the EFA, Bartlett's test of sampling adequacy and Kaiser–Meyer–Olkin were obtained. The exploratory factor analysis was performed using Varimax rotation, resulting in the formation of rotated component matrices for the three subscales. Test–retest reliability of the translated scales was explored at a 1-week interval. The analysis reveals that the test–retest value was estimated between 0.736 and 0.943, which represents the value > 0.70 is more reliable and valid.

Confirmatory factor analysis (CFA) was performed to estimate the model fitness and overall consistency of the model on which the psychological measure is based. CFA revealed significant values for different model fit indices, like Goodness of Fit Index (GFI) and Adjusted GFI, Normative Fit Index (NFI), Relative Fit Index (RFI), Tucker–Lewis Index (TLI), Comparative Fit Index (CFI), and Root Mean Square Error of Approximation (RMSEA). With significant exploratory and confirmatory factor analysis results, phase II of the study was completed. The third phase consisted of estimating the validity of the translated scales with already existing standardized psychological assessment measures. The major validity assumptions were formulated.

Results suggest that the use of inhibition and dysregulated expression, which are both maladaptive emotion regulation strategies, was significantly positively related to the shy behavior and the use of emotional suppression to manage anger, worry, and sadness emotions (57). Therefore, it indicates that children who have shyness as a dominant personality trait exhibited the use of inhibition and dysregulated expression more than children who have a lesser score on the shyness scale (58). Additionally, a significant positive correlation was observed between the aforementioned maladaptive emotion regulation strategies (inhibition and dysregulated expression) and the three subscales of the School Burnout Inventory (59). This suggests that an unhealthy emotion regulation choice is significantly related to exhaustion and burnout in children at school (60).

It was observed that children who use unhealthy emotion regulation strategies like inhibition and dysregulated expression showed a lack of cognitive reappraisal strategy in a time of an emotional crisis (61). Additionally, these children also showed a low score on the distress tolerance and self-esteem scale, which further signifies the use of a positive emotion regulation strategy to promote a healthy lifestyle and a positive self-image. An interesting finding of the correlational analysis suggested that parenting style significantly affects the choice of emotion regulation strategy chosen by children. A higher score on the scale of parenting style was negatively related to the use of maladaptive emotion regulation strategies (62).

The results indicate that children who use positive emotion regulation strategies like emotional coping have a higher distress tolerance level and more frequently use cognitive reappraisal strategy during an emotional crisis (63). Additionally, the use of emotional coping was found to be significantly positively related to a higher self-esteem score, thereby eventually predicting a healthy life approach. In terms of child–parent relationships, these correlational analyses significantly linked healthy parenting style to the choice of healthy emotion regulation strategy. The results indicate that children who chose emotional coping as an emotion regulation strategy had a low school burnout score. Additionally, correlational analyses suggested that emotional coping was less observed in shy children and in those who displayed the use of emotional suppression in times of emotion management (64). Significant correlation values were obtained favoring all the study assumptions, thereby highlighting the validity of the scales. This resulted in the completion of the study.

### Limitations of the study

In this study, the CEMS was translated and validated into Urdu language, as there was a need to assess emotions in our children through a reliable and valid scale. This was the main strength of this study, while on the other hand, the study has some limitations. This study is validated over normal children who never experienced any kind of emotional disturbance in the past. So, the scale used is not preferably useful for those children who have psychiatric symptoms and physical or intellectual disabilities. This study was conducted with specific age groups of children not on the children of all age groups.

## Implications of the study

CEMS was translated and validated into the Urdu language in the current study; this will enable practitioners from diverse backgrounds to use this set of scales to better gauge children's psychopathological tendencies. Knowing about the emotional management prowess of children is important for teachers, social workers, psychiatrists, and psychologists working with children in order to make better management and treatment plans for the young ones as their sadness, anger, and worry levels are very important when making such plans (38).

## Data availability statement

The raw data supporting the conclusions of this article will be made available by the authors, without undue reservation.

## Ethics statement

The studies involving humans were approved by the Institutional Review Board (IRB), Government College University Faisalabad. The studies were conducted in accordance with the local legislation and institutional requirements. Written informed consent for participation in this study was provided by the participants legal guardians/next of kin.

## Author contributions

All authors listed have made a substantial, direct, and intellectual contribution to the work and approved it for publication.
